# Obituary

**Published:** 1850-08

**Authors:** 


### OBITUARY.

The numerous admirers of Dr. John T. Shotwell, late Professor of Anatomy in the Medical College of Ohio, will be grieved to learn that he died in Cincinnati, on July, 1850. His disease was cholera.

We knew Dr. Shotwell during his collegiate life, and while he was engaged in the study of medicine. His fine powers of mind, and studious habits held out the promise of professional distinction, and he speedily attained eminence as a practitioner of medicine in the city of Cincinnati. Upon the resignation of Professor Cobb, of the Chair of Anatomy in the Medical College of Ohio, Dr. Shotwell was called upon by the trustees to fill the place, and he discharged the duties to the satisfaction of his classes, up to the time of his death.

As a practitioner of medicine, Dr. Shotwell was skillful and successful; as a teacher he was eminently respectable, and as a friend and companion, he was all that was pleasing and charming. His conversational gifts were of a high order, and as a humorist he was almost equal to Dr. Arberthnot, the companion of Pope and Swift. The social circle in which Professor Shotwell moved will long deplore his loss, and few can be found worthy to fill his professional position.

Dr. Shotwell was forty-three years of age, and when we saw him a few weeks ago, at the Medical Association, he seemed to have the promise of a long life before him. In the midst of his usefulness, he is cut down, but his virtues will long be cherished in the memories of those who knew him. B.

